# Anti-fibrotic effects of rosmarinic acid on Tenon’s capsule fibroblasts stimulated with TGF-β: therapeutic potential in ocular surgery

**DOI:** 10.5935/0004-2749.20200042

**Published:** 2020

**Authors:** Carolina Maria Módulo, Larissa Domenegueti Ferreira, Lilian Eslaine Costa Mendes da Silva, Marco Andrey Cipriani Frade, Peter Sol Reinach, Eduardo Melani Rocha, Jayter Silva Paula

**Affiliations:** 1 Department of Ophthalmology, Otorhinolaryngology and Head and Neck Surgery, Faculdade de Medicina de Ribeirão Preto, Universidade de São Paulo, Ribeirão Preto, Brazil; 2 Dermatology Division, Department of Clinical Medicine, Faculdade de Medicina de Ribeirão Preto, Universidade de São Paulo, Ribeirão Preto, Brazil; 3 Department of Ophthalmology and Optometry, Wenzhou Medical University, Wenzhou, PR China

**Keywords:** Glaucoma, Ophthalmologic surgical procedures, Fibroblasts, Healing, Rosmarinic acid, Glaucoma, Procedimentos cirúrgicos oftalmológicos, Fibroblastos, Cicatrização, Ácido rosmarínico

## Abstract

**Purpose:**

Collagen deposition and myofibroblast differentiation are critical factors related to excessive scarring in ocular surgeries. This study evaluated the anti-fibrotic activity of rosmarinic acid on rabbit Tenon’s capsule fibroblasts stimulated with transforming growth factor-b2.

**Methods:**

Primary cultures of rabbit Tenon’s capsule fibroblasts were treated with various concentrations of rosmarinic acid for 12 h, in the presence and absence of transforming growth factor-b2. After 48 h, the proliferation index of rabbit Tenon’s capsule fibroblasts and the differentiation of myofibroblasts were investigated through immunofluorescence staining for proliferating cell nuclear antigen and alpha smooth muscle actin. An automated cell counter and colorimetric metabolic activity assay were used to evaluate cell number and viability. Collagen expression and production were determined by quantitative real-time polymerase chain reaction and hydroxyproline assay, respectively.

**Results:**

Unstimulated rabbit Tenon’s capsule fibroblasts treated with any concentration of rosmarinic acid exhibited diminished collagen expression (p<0.01) but showed no differences in proliferation index. Transforming growth factor-b2 exposure induced myofibroblast differentiation and increased collagen production. Exposure to rosmarinic acid at 1.0 and 3.0 µM concentrations reduced the proliferation index (p<0.02), as well as the collagen expression and hydroxyproline content (p<0.05). Exposure to 3.0 µM rosmarinic acid reduced viability (p=0.035) in unstimulated rabbit Tenon’s capsule fibroblasts and cell numbers (p=0.001) in both stimulated and unstimulated rabbit Tenon’s capsule fibroblast cultures.

**Conclusions:**

Exposure to 1.0 µM rosmarinic acid was noncytotoxic and led to reduced collagen expression and proliferation of stimulated rabbit Tenon’s capsule fibroblasts. These findings suggest that rosmarinic acid is a relatively non-injurious anti-fibrotic compound to rabbit Tenon’s capsule fibroblasts, with potential application as an adjunctive agent in ocular procedures, particularly in glaucoma surgeries.

## Abstract (pt)

**Objetivo:**

A deposição de colágeno e a diferenciação de miofibroblastos são fatores chaves re lacio nados à cicatrização excessiva em cirurgias oculares. Este estudo avaliou a atividade anti-fibrótica do ácido rosmarínico nos fibroblastos da cápsula de Tenon de coelhos estimulados com o fator de crescimento transformador-b2.

**Métodos:**

Culturas primárias de fibroblastos da cápsula de Tenon de coelhos foram tratadas com várias concentrações de ácido rosmarínico por 12h, na presença e na ausência do fator de crescimento transformador-b2. Após 48h, o índice de proliferação dos fibroblastos da cápsula de Tenon de coelhos e a diferenciação dos miofibroblastos foram investigados por coloração por imunofluorescência para proliferação de antígeno nuclear celular e α-actina de músculo liso, respectivamente. Um contador automático de células e um ensaio de atividade metabólica colorimétrica foram utilizados para avaliar o número e a viabilidade das células. A expressão e produção do colágeno foram determinadas por reação quantitativa em cadeia da polimerase em tempo real e ensaio de hidroxiprolina, respectivamente.

**Resultados:**

Fibroblastos da cápsula de Tenon de coelhos não estimulados tratados com qualquer concentração de ácido rosmarínico exibiram diminuição de colágeno (p<0,01), mas não mostraram diferenças no índice de proliferação. A exposição ao fator de crescimento trans formador-b2 induziu a diferenciação de miofibroblastos e aumentou a pro dução de colágeno. A exposição ao ácido rosmarínico nas concentrações de 1,0 e 3,0 µM reduziu o índice de proliferação (p<0,02), bem como a expressão de colágeno e a quantificação de hidroxiprolina (p<0.05). A exposição a 3,0 µM de ácido rosmarínico reduziu a viabilidade (p=0,035) de fibroblastos da cápsula de Tenon de coelhos não estimulados e o número de células (p=0,001) em culturas de fibroblastos da cápsula de Tenon de coelhos estimuladas e não estimuladas.

**Conclusões:**

A exposição ao ácido rosmarínico 1,0 µM foi não citotóxica e levou à expressão reduzida de colágeno e menor proliferação de fibroblastos da cápsula de Tenon estimulados pelo fator de crescimento transformador-b2. Esses achados sugerem que o ácido rosmarínico é um composto antifibrótico relativamente não lesivo aos fibroblastos da cápsula de Tenon de coelhos, com potencial aplicação como agente adjuvante em procedi mentos oculares, particularmente em cirurgias de glaucoma.

## INTRODUCTION

Some ocular surgeries, such as glaucoma fistulizing surgeries, depend on a highly coordinated conjunctival healing process. Trabeculectomy is the most common surgical procedure in glaucoma patients used to reduce both the intraocular pressure (IOP) and the rate of visual field deterioration, when medical treatment fails^(1-4)^. After surgery, IOP reduction is achieved by draining the aqueous humor to the subconjunctival space, forming a filtering bleb interspersed within Tenon’s capsule^(5)^.

The major cause of trabeculectomy failure is the formation of a dense fibrotic material in the subconjunctival space, due to enhanced fibroblast proliferation, collagen deposition, and neovascularization of the surgical site^(5,6)^. During postoperative healing, Tenon’s capsule fibroblasts undergo activation by local cytokines and growth factors, such as transforming growth factor-b (TGF-β)^(7)^. Some of these fibroblasts can be induced by TGF-β to differentiate into myofibroblasts. Both fibroblasts and myofibroblasts contribute to this process through paracrine interactions, thereby eliciting collagen production and extracellular matrix remodeling^(8,9)^.

Thus, the reduction of surgically induced excessive scarring during wound healing with anti-fibrotic agents is crucial for sustained improvement of aqueous humor drainage, as well as good surgical outcomes. Mitomycin C and 5-fluorouracil are current first-line healing modulators and are the most frequently used adjunctive therapies in routine trabeculectomy^(10,11)^. However, their usage is associated with several postoperative complications, such as chronic filter bleb leakage, hypotonia, and devastating ocular infections^(10-14)^.

Rosmarinic acid (RA) is a natural phenolic compound commonly found in *Boraginaceae* species^(15)^. It has several biological activities, including antioxidative, antitumoral, anti-inflammatory, anti-fibrotic, and anti-angiogenic actions in different tissues^(15,16)^. We previously showed that RA exhibits transient anti-neovascularization effects after glaucoma filtration surgery in rabbits^(16)^; here, we evaluated whether RA also suppresses TGF-β-induced fibrosis mediated by rabbit Tenon’s capsule fibroblasts (RTFs). We aimed to determine if RA could be used as a novel therapeutic alternative for improving the outcome of glau coma filtration surgeries.

## METHODS

### Cell culture

Twenty-four Tenon’s capsule samples from eight eyes of four adult male New Zealand rabbits (approximately 10 mg/animal) were biopsied from the superior quadrant through 180° peritomies of both eyes. This experimental *in vitro* study followed the Association for Research in Vision and Ophthalmology ethical standards and rules for testing animals; all procedures were approved by the ethics committee on Animal Experimentation of Ribeirão Preto Medical School, Universidade de São Paulo (#124/2009). After samples were collected, they were washed and placed in tubes containing 3.0 ml of Hanks balanced salt solution (Gibco, Life Technologies Corporation, New York, NY, USA). Under aseptic conditions, Tenon’s capsule was carefully fragmented with sterile surgical forceps and scissors, and the pieces were placed in six-well plates containing Dulbecco’s Modified Eagle Medium supplemented with penicillin 100 IU/ml, streptomycin 100 µg/ml, and 10% fetal bovine serum (FBS, Sigma-Aldrich Co., St. Louis, MO, USA), at 37°C with 5% CO2^(7)^.

### TGF-β2 exposure

Third-passage RTFs (15 to 25 days after extraction) were seeded in triplicate in 24-well plates and maintained until they reached 60% confluence. Then, medium with inactivated FBS, with or without 5.0 ng TGF-β2 (recombinant human TGF-β2; R&D Systems Inc., SP, Brazil), was added to the plates, and cells were cultured for 48 h.

### Rosmarinic acid exposure

Regardless of the presence or absence of TGF-β2, cells were exposed to inactivated FBS with or without 0.3, 1.0, or 3.0 µM RA (Sigma-Aldrich Co.) for 12 h. Subsequently, the medium was replaced with drug-free inactivated FBS medium, and the RTFs were maintained for an additional 48 h.

### Cell counting

The effects of different concentrations of RA treatment on the numbers of live cells were studied by cell counting using flow cytometry. Prior to cell counting, the medium was discarded, and the plates were washed with phosphate-buffered saline; they were then incubated with 300 µL of 0.25% Trypsin-EDTA (Gibco, Life Technologies Corporation). After the addition of 300 µL of inactivated FBS medium, samples were transferred to Eppendorf tubes and centrifuged for 5 min at 1,000 rpm. Cell pellets were suspended in 10 µL of 0.4% Trypan Blue Staining Solution (Gibco, Life Technologies Corporation), resulting in a dilution factor of 1. Stained cells were then quantified with the Countess Automated Cell Counter™ protocol (Invitrogen Corporation, Carlsbad, CA, USA).

### MTT assay for cell viability

The 3-(4,5-dimethylthiazol-2-yl)-2,5-diphenyltetrazolium bromide (MTT) colorimetric assay was used to assess RTF viability. The medium was first discarded, and 300 µL of fresh medium containing 5 mg/ml MTT was added. Plates were then incubated for 3 h in the dark, at 37°C with 5% CO2, to allow formazan crystal formation. Subsequently, the well contents were solubilized with 300 µL of dimethyl sulfoxide. The absorbance at 570 nm was measured with the Varian Cary 50 spectrophotometer (Varian Inc., Agilent Technologies); the readings were interpreted as normalized percentages of control values.

### Immunofluorescence assays

Anti-alpha-SMA and anti-proliferating cell nuclear antigen (PCNA) antibody immunostaining assays were used to evaluate myofibroblast differentiation and the proliferation index (PI), respectively. Cells were cultured on coverslips using the abovementioned protocol. Subsequently, they were incubated in blocking solution with monoclonal primary anti-alpha-SMA antibody 1:100 (Ab181407, Abcam plc., Cambridge, UK) or anti-PCNA (PC10) antibody 1:100 (2586, Cell Signaling Technologies Inc., Danvers, MA, USA) for 4 h, followed by exposure to the secondary antibody Alexa Fluor^®^ 488 1:1000 (A21202, Life Technologies, Carlsbad, CA, USA) for another 30 min at room temperature. Slides were mounted with ProLong^®^ Gold antifade reagent with DAPI (P36931, Life Technologies) and photographed in a microscope (Leica, Wetzlar, Germany) equipped with a digital camera. The PI is presented as a percentage by obtaining the number of cells with well-defined nuclear anti-PCNA staining and dividing this number by the total number of cells (´100) per visual field. One of the authors (CMM, who was blinded to the sample identities) counted the cells on all sample coverslips and performed the calculations. For semiquantitative analysis of myofibroblasts, the proportion (%) of cells with strong anti-alpha-SMA staining and well-organized fibers, relative to the overall RTF number per microscopic field, was also counted and calculated for each group.

### Semiquantitative collagen type I RT-PCR

Samples of the fibroblasts were frozen and stored in microtubes containing RNAprotect Cell Reagent (QIAGEN Biotecnologia Brasil Ltda, SP, Brazil) and underwent total RNA extraction using the RNeasy Micro Kit (QIAGEN Biotecnologia Brasil Ltda). cDNA was obtained by performing reverse transcription with 100 ng of total RNA using the QuantiTect Reverse Transcription Kit (QIAGEN Biotecnologia Brasil Ltda). Collagen type 1 mRNA expression was quantified with the BIO-RAD iQ5 PCR System (Bio-Rad, Hercules, CA, USA), using the TaqMan Gene Expression assay for *COL1A1* (Assay ID: Oc03396074_g1; Applied Biosystems, Foster City, CA, USA), followed by incubation with Taq polymerase (TaqMan Universal PCR Master Mix, No AmpErase UNG-2X, Applied Biosystems). We used *GAPDH* as the endogenous control reference gene (Assay ID: Oc03823402_g1, Applied Biosystems). Differential gene expression was evaluated by using the 2^(-DDC(t))^ methodology to calculate fold changes in *COL1A1* gene expression, normalized to *GAPDH* level.

### Colorimetric hydroxyproline assay

Triplicate samples from RTF cultures were used for the indirect evaluation of collagen production by measurement of hydroxyproline levels. All cell cultures received the same treatment protocol; however, supernatant samples were collected after replacement of media with drug-free inactivated FBS, and RTF cultures were maintained for an additional 72 h. Hydroxyproline contents were quantified using a Hydroxyproline Colorimetric Assay Kit (MAK008, Sigma-Aldrich), in accordance with the manufacturer’s instructions.

### Statistical analysis

All experiments were performed at least three times, using triplicates for each treatment group. Data are expressed as means, percentages, and standard errors. The unpaired Student’s t-test was used for comparisons of continuous well-controlled variables between groups. Fisher’s exact test was performed to compare semiquantitative results of alpha-SMA staining. Statistical analyses were performed with Prism software, version 5.0 (GraphPad Software Inc., La Jolla, CA, USA). P-values of <0.05 were considered statistically significant.

The datasets analyzed during the current study are available from the corresponding author on reasonable request.

## RESULTS

**Figure 1:**
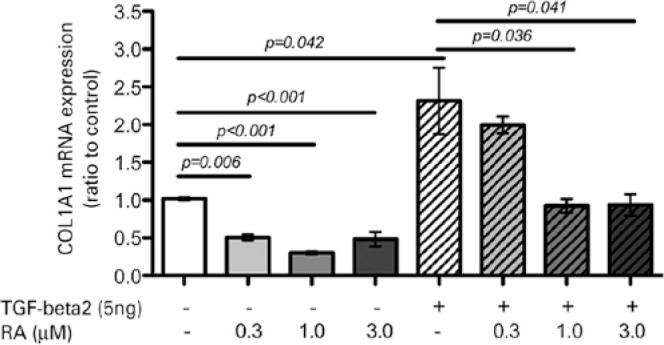
Relative expression of collagen alpha I type I (*COL1A1*) in RTFs stimulated or not with TGF-β2 and treated or not with 0.3, 1.0, and 3.0 µM RA for 12 h. *GAPDH* was used as the endogenous control reference gene.

**Figure 2:**
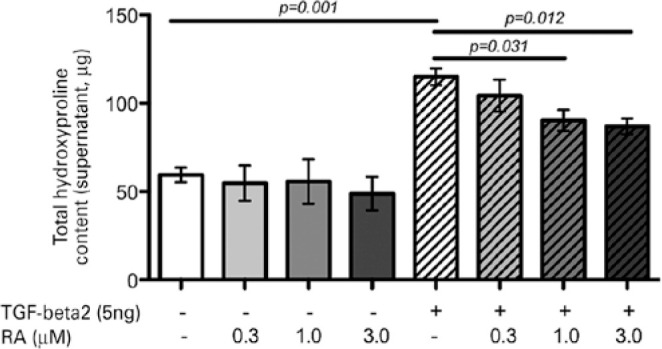
Total hydroxyproline content obtained from supernatants collected after 72 h from cultured RTFs stimulated or not with TGF-β2 and treated or not with 0.3, 1.0, and 3.0 µM RA for 12 h. The hydroxyproline assay was used to estimate total collagen production in the samples.

**Figure 3:**
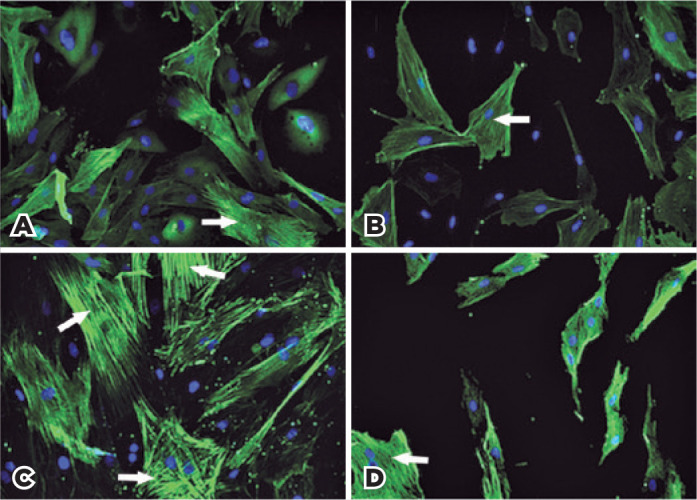
Alpha-SMA immunofluorescence of unstimulated RTFs (A and B) and stimulated RTFs (C and D), treated (B and D) or not (A and C) with 1.0 µM RA. Note the increased staining of organized α-SMA under TGF-β2 stimulation (C). Treatment with 1.0 µM RA (B and D) induced a reduction in the amount and orientation of fibers in stimulated and unstimulated cells. White arrows indicate the strong, well-organized alpha-SMA fibers attributed to myofibroblasts, which were more frequently observed in TGF-β2-stimulated cells.

After 48 h of exposure to TGF-β2, both RTF cell viability and proliferation were unchanged. In contrast, relative *COL1A1* gene expression increased twofold (p=0.042; Figure 1), as did hydroxyproline content (p=0.001; Figure 2). These increases were accompanied by myofibroblast differentiation, evidenced by increases in the numbers of cells with robust increments in organized intracellular alpha-SMA expression (mean proportions of cells with strong anti-alpha-SMA staining: 31.3% [unstimulated RTFs] versus 41.5% [stimulated RTFs]). Treatment with 1.0 µM RA was associated with a significant reduction in the proportion of myofibroblasts (25.4% [treated RTFs] versus 14.1% [untreated RTFs], p=0.04; Figure 3).

*COL1A1* mRNA expression levels were also lower in cells treated with any concentration of RA, but the reduction was not significant in TGF-β2-stimulated RTFs treated with 0.3 µM RA (Figure 1). Hydroxyproline levels were also significantly reduced in stimulated cells treated with both 1.0 and 3.0 µM concentrations of RA (Figure 2).

**Figure 4:**
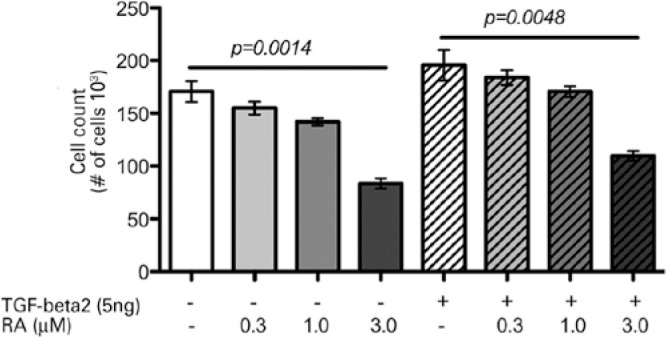
Cell counting of RTFs stimulated with or without TGF-β2 and treated or not with 0.3, 1.0, and 3.0 µM RA for 12 h, compared with their respective controls.

**Figure 5:**
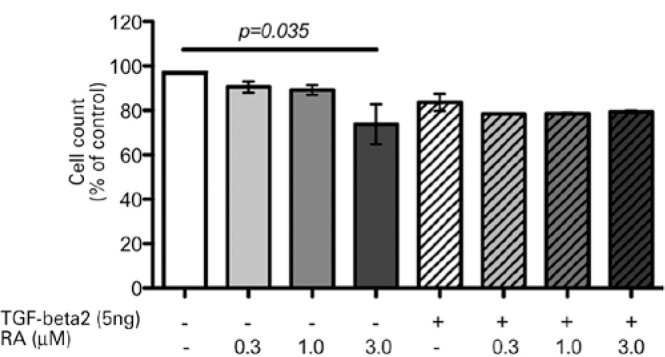
MTT analysis of cell viability of RTFs stimulated or not with TGF-β2 and treated or not with 0.3, 1.0, and 3.0 µM RA for 12 h, compared with their respective controls.

**Figure 6:**
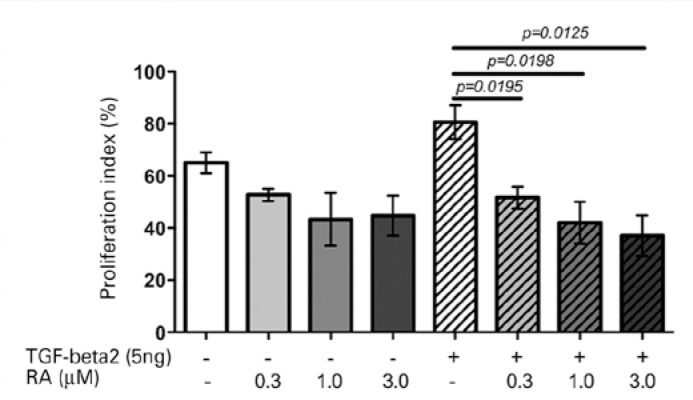
Proliferation index calculated by the proportion of anti-PCNA-stained RTFs. Cells stimulated or not with TGF-β2 and treated or not with 0.3, 1.0, and 3.0 µM RA for 12 h were compared with their respective controls.

With either 0.3 or 1.0 µM concentration of RA, the results of cell counting and MTT assays indicated no significant reductions in cell viability, regardless of the presence or absence of TGF-β2 (Figures 4 and 5). However, 3.0 µM RA exhibited a degree of cytotoxicity, because this concentration of RA reduced the numbers of stimulated cells by 44% (p=0.0048) and unstimulated cells by 51% (p=0.0014). Similarly, changes in the MTT absorbance values showed reduced viability of approximately 24% (p=0.035) in unstimulated cells exposed to 3.0 µM RA (Figures 4 and 5). The PI also declined at all RA concentrations in RTFs exposed to TGF-β2. The reductions of anti-PCNA-stained cell number reached 54% (p<0.02), relative to the values observed in unstimulated cells (Figure 6).

## DISCUSSION

Excessive fibrosis during wound healing is the main factor in the surgical failures that are frequently observed in glaucoma fistulizing procedures^(5,6,17,18)^. As previously mentioned, no modulatory agents can be cli nically used to inhibit postoperative fibrosis after glaucoma surgery without additional induction of severe ocular complications^(12-14)^. Following surgically related induction of inflammation, fibrosis is eventually attributable to fibroblast activation and differentiation into myofibroblasts. These changes, which are induced by TGF-β sig naling activation, also underlie extracellular matrix remodeling associated with the accumulation of fibrosis-related extracellular matrix proteins^(7,19-23)^.

Previous reports have demonstrated the anti-fibrotic actions of RA^(24-26)^, including its anti-angiogenic effects in the conjunctiva^(16)^; here, we demonstrated comparable effects of nontoxic RA concentrations in RTF cultures.

Various cytokines and local factors are upregulated at surgical sites in the subconjunctival space^(27)^. As the local fibrotic effect includes increases in TGF-β2 levels^(28,29)^, we mimicked this effect *in vitro* by exposure of RTFs to TGF-β2. Under this condition, a robust increase occurred in fibrillar alpha-SMA content and stress-like organization, along with upregulation of *COL1A1* mRNA expression and synthesis of collagen, both of which are biomarkers of myofibroblast differentiation. Moreover, the nonsignificant increases in cell viability or proliferation following TGF-β exposure, which were described in previous studies^(30)^, could have been related to protocol differences, such as the cell confluence used in each study. Our demonstration of myofibroblast differentiation is consistent with increases in the expression and production of collagen, as well as in the alpha-SMA protein staining activity, factors regarded as biomarkers of cellular differentiation^(8,22,31-33)^.

Although 3.0 µM RA most effectively suppressed myofibroblast differentiation and collagen 1 gene expression, its inhibitory effects on viability and PI may also be attri butable to RA cytotoxicity in both stimulated and unstimulated RTFs. Nonetheless, neither 0.3 nor 1.0 µM RA could be considered cytotoxic, as RTF counts and viability did not reveal significant reductions. Based on MTT viability results, we speculate that both stimulated and unstimulated RTFs may not be metabolically disturbed by 0.3 or 1.0 µM RA^(34)^. We acknowledge potential bias related to the possible presence of leftover hydroxyl species contaminants in cultures treated with RA during the MTT protocol. As previously shown^(35)^, some phenolic compounds can reduce MTT in the absence of live cells, which could explain the lack of consistency between the results of cell counting and MTT viability assays. Although the results were not significant in unstimulated RTFs, the reduced PI may have also been influenced by the total number of live cells, which was lower in the group treated with 3.0 µM RA. However, we presume that despite the loss of dead cells during the immunostaining process, the reduced PI may demonstrate the inhibition of proliferation by RA.

Notably, 1.0 µM RA may be an appropriate target concentration for further studies, as RTF viability was unchanged at this concentration of RA; concurrently, 1.0 µM RA induced reductions in alpha-SMA content, PI, *COL1A1* expression, and hydroxyproline levels (indicative of collagen production) in TGF-β2-stimulated RTFs. Any assumption of the noncytotoxic effect of 1.0 µM RA requires further *in vivo* tests considering the presented limited approach used-a short-term *in vitro* exposure of cultured cells.

Suppression of both myofibroblast differentiation and extracellular matrix collagen deposition has been proposed as objective for controlling subconjunctival fibrosis^(29,31-33)^. Although phenolic components have shown several beneficial anti-fibrotic actions in other tissues, including inhibition of myofibroblast differentiation, and collagen synthesis^(24-26,33)^, the present study is the first to extend those findings by showing that RA exposure suppresses myofibroblast differentiation and collagen expression in ocular fibroblasts. These results support additional evaluation of the possible use of RA in a clinical setting, because they complement our previous finding that RA has anti-angiogenic effects in an experimental glaucoma surgery model^(16)^.

Previous preliminary *in vivo* tests showed no significant differences in collagen deposition based on the experimental surgical site, which we attribute to a po tential interplay of resident cells other than local fibroblasts^(16)^. Taken together, the selective anti-fibrotic and anti-angiogenic effects of stimulation with ≤1 µM RA should prompt further clinical studies of its safety, efficacy, and local bioavailability for adjunctive use in several ocular surface procedures, such as glaucoma fistulizing surgery. Upcoming tests should also evaluate both the beneficial and adverse effects of RA on all ocular tissues, particularly on the ocular surface.
